# Supplementary material for: Spectral Analysis of the QT Interval Increases the Prediction Accuracy of Clinical Variables in Brugada Syndrome
Source: J Clin Med. 2019 Oct 4;8(10):1629. doi: 10.3390/jcm8101629 (PMC6833061; doi:10.3390/jcm8101629)
Supplement: Supplementary file 1 [file jcm-08-01629-s001.pdf]

Spectral analysis of the QT interval increases prediction accuracy of clinical variables in the Brugada Syndrome

García-Iglesias D, et al.

SUPPLEMENTAL MATERIAL

Supplemental Methods

1. Extraction of QRS complexes using a modified Pan-Tompkins algorithm

The standard ECGs (12 leads) of the patients included in the study were digitally collected for at least 12 consecutive seconds (1 KHz sample rate; 12 bit resolution; Band-pass filtered 0.05-150 Hz; EPTracer® v1.05.v3, CardioTek) (Figure S2). To extract the QRS complexes, we propose a modified algorithm of the originally described by Pan-Tompkins[1]. As previously described[2], the latter consist on four consecutive steps: differentiation, squared elevation, detection threshold calculation and correction by local maxima. Thereafter, we introduced some modifications as follows (see also figure S2):

- First, we normalized the ECG signal to allow appropriate comparisons between leads and patients.
- In order to determine the threshold for detection of the R-wave in the QRS complexes (after differentiation and squared elevation of the signal), the 99.5 percentile of the signal (*P99.5*) was calculated. All the extreme values (defined as those greater than *P99.5*) were removed and the signal was typified over the value of *P99.5* (Figure S2B). A threshold of 0.6 defined the time points where there were QRS complexes (*tQRS*; red line in Figure S2B).
- A temporal correction is performed for the morphology of the QRS complexes. To do this, the point of the QRS complex with a higher positive voltage value in the V6 derivation was selected (*tMaxQRS*; red line in Figure S2C), because clinically it is the lead in which the peak of the R wave is better defined.
- Finally, to subtract the ST complexes for analysis a window of 450 ms around the *tMaxQRS* point was selected (from 60 ms before *tMaxQRS* to 390 ms after *tMaxQRS*; Figure S2C).

Once the QRS peak was identify, a 450 ms window was selected (60 ms before and 390 ms after the QRS peak) to extract the QT complex (Figure S2). The QT interval was later divided in QRS interval (first 120 ms) and ST-Twave interval (last 330 ms).

2. Wavelet continuous transformation for the analysis of the high-frequency content

The time-frequency data of each QT complex were collected using the Wavelet transform (Morlet wavelet)[3]. As previously described, data were analyzed in the defined range of high frequencies (85-130 Hz)[3], with an upper period of 11.5 ms and lower period of 7.7 ms. A temporal definition of 1 kHz and a frequency resolution of 1/125 suboctaves were used. Calculations for the Wavelet Continuous Transform were performed with the WaveletComp library for R[4].

Supplementary references

1. Pan J, Tompkins WJ: A Real-Time QRS Detection Algorithm. IEEE Transactions on Biomedical Engineering 1985; BME-32:230–236.

2. García Iglesias D, Roqueñi Gutiérrez N, De Cos JF, Calvo D: Analysis of the High-Frequency Content in Human QRS Complexes by the Continuous Wavelet Transform: An Automatized Analysis for the Prediction of Sudden Cardiac Death. Sensors 2018; 18:560.

3. Morlet J, Arens G, Fourgeau E, Giard D: Wave propagation and sampling theory—Part II: Sampling theory and complex waves. Geophysics 1982; 47:222–236.

4. Roesch A, Schmidbauer H: WaveletComp: Computational Wavelet Analysis [Internet]. 2018,. Available from: https://CRAN.R-project.org/package=WaveletComp

Supplementary tables

Table S1

	Type I ECG	Type II or III ECG	Normal ECG	p
All precordial leads				
**Peak Power**	0.629 (0.421 - 0.836)	1.518 (0.186 - 2.85)	1.252 (0.212 - 2.292)	0.604
**Total Power**	47.415 (20.269 - 74.561)	69.721 (28.191 - 111.251)	39.834 (16.025 - 63.643)	0.729
**Total QRS Power**	16.665 (11.358 - 21.972)	38.651 (5.058 - 72.244)	27.283 (6.545 - 48.021)	0.606
**Total ST Power**	30.75 (7.171 - 54.329)	31.07 (16.856 - 45.283)	12.551 (7.645 - 17.457)	1
**QRS to ST Total Power**	3.849 (2.131 - 5.566)	5.853 (3.926 - 7.779)	7.076 (3.14 - 11.013)	0.468
Right precordial leads				
**Peak Power**	0.886 (0.529 - 1.244)	1.948 (0.43 - 3.466)	0.978 (0.447 - 1.509)	0.555
**Total Power**	89.832 (17.724 - 161.941)	120.243 (59.55 - 180.936)	41.584 (24.785 - 58.383)	0.785
**Total QRS Power**	25.041 (15.957 - 34.126)	53.695 (13.362 - 94.027)	24.309 (11.623 - 36.994)	0.544
**Total ST Power**	64.791 (0.574 - 129.008)	66.549 (31.128 - 101.969)	17.275 (10.986 - 23.564)	0.998
**QRS to ST Total Power**	3.471 (1.768 - 5.173)	4.284 (2.783 - 5.785)	4.275 (1.633 - 6.917)	0.807

Table S1. Comparative analysis of the High Frequency Content between different ECG patterns in BrS patients. Within brackets is denoting the CI95%. Units for Peak Power, Total Power, Total QRS Power and Total ST Power are expressed as $10^3 nV^2 Hz^{-1}$.

Table S2

	BrS Patients	NR patients	p
All precordial leads			
Peak Power	1.252 (0.212 - 2.292)	0.732 (0.546 - 0.919)	0.324
Total Power	39.834 (16.025 - 63.643)	29.29 (24.158 - 34.421)	0.384
Total QRS Power	27.283 (6.545 - 48.021)	17.928 (13.788 - 22.068)	0.374
Total ST Power	12.551 (7.645 - 17.457)	11.362 (8.491 - 14.232)	0.674
QRS to ST Total Power	7.076 (3.14 - 11.013)	8.036 (4.489 - 11.584)	0.716
Right precordial leads			
Peak Power	0.978 (0.447 - 1.509)	0.713 (0.476 - 0.95)	0.36
Total Power	41.584 (24.785 - 58.383)	37.103 (28.495 - 45.712)	0.633
Total QRS Power	24.309 (11.623 - 36.994)	20.158 (13.903 - 26.413)	0.555
Total ST Power	17.275 (10.986 - 23.564)	16.945 (11.654 - 22.236)	0.936
QRS to ST Total Power	4.275 (1.633 - 6.917)	4.871 (3.377 - 6.365)	0.693

Table S2. Comparative analysis of the High Frequency Content between normal ECG patterns recorded from different clinical conditions. Within brackets is denoting the CI95%. Units for Peak Power, Total Power, Total QRS Power and Total ST Power are expressed as $10^3 nV^2 Hz^{-1}$. NR: Negative responders.

Table S3

	Tipo 2			Tipo 3		
	BrS Patients	NR Patients	p	BrS Patients	NR Patients	p
	N=62	N=36		N=22	N=39	
**All Precordial Leads**						
Peak Power	1.815 (0.008 - 3.623)	1.099 (0.629 - 1.57)	0.446	0.679 (0.305 - 1.054)	1.043 (0.618 - 1.468)	0.194
Total Power	83.952 (27.928 - 139.976)	40.065 (26.031 - 54.1)	0.133	29.615 (14.949 - 44.281)	32.744 (20.768 - 44.721)	0.735
Total QRS Power	46.531 (0.944 - 92.117)	26.878 (15.491 - 38.264)	0.405	16.445 (7.511 - 25.379)	24.148 (15.245 - 33.052)	0.216
Total ST Power	37.421 (18.607 - 56.235)	13.188 (8.375 - 18)	**0.015**	13.17 (2.699 - 23.641)	8.596 (3.294 - 13.898)	0.426
QRS to ST Ratio	5.877 (3.419 - 8.335)	6.694 (2.953 - 10.435)	0.713	5.783 (3.007 - 8.56)	17.151 (5.848 - 28.454)	0.054
**Right Precordial Leads**						
Peak Power	2.394 (0.338 - 4.451)	1.471 (0.761 - 2.18)	0.398	0.689 (0.365 - 1.013)	0.967 (0.416 - 1.519)	0.379
Total Power	149.305 (67.926 - 230.684)	65.494 (38.444 - 92.544)	0.054	38.343 (22.091 - 54.594)	38.934 (19.05 - 58.818)	0.963
Total QRS Power	66.228 (11.619 - 120.837)	40.437 (19.769 - 61.104)	0.379	18.374 (9.17 - 27.578)	25.668 (12.935 - 38.402)	0.347
Total ST Power	83.077 (35.619 - 130.535)	25.057 (13.058 - 37.056)	**0.021**	19.968 (7.673 - 32.264)	13.265 (4.433 - 22.098)	0.367
QRS to ST Ratio	4.469 (2.546 - 6.391)	5.583 (2.002 - 9.163)	0.581	3.764 (1.678 - 5.85)	9.589 (2.32 - 16.859)	0.125

Table S3. Comparative analysis of the High Frequency Content between type 2 and type 3 Brugada pattern recorded from different clinical conditions. Within brackets is denoting the CI95%. Units for Peak Power, Total Power, Total QRS Power and Total ST Power are expressed as $10^3 nV^2Hz^{-1}$. NR: Negative responders.

Table S4

	Induct-BrS Patients	NR patients	p value	Flecainide	Ajmaline	p value
Attenuation observed in all precordial leads						
**Peak Power (Absolute)**	0.508 (0.157)	0.412 (0.06)	0.591	0.388 (0.066)	0.584 (0.116)	0.159
**Peak Power (%)**	34.343 (4.45)	29.528 (2.925)	0.395	31.023 (2.562)	31.236 (5.361)	0.972
**Total Power (Absolute)**	17.059 (6.041)	10.52 (1.788)	0.332	11.165 (2.281)	15.965 (3.994)	0.317
**Total Power (%)**	20.825 (4.259)	21.611 (3.124)	0.888	21.264 (2.752)	24.721 (4.766)	0.546
**Total QRS Power (Absolute)**	11.737 (4.002)	9.266 (1.232)	0.58	9.04 (1.582)	12.285 (2.288)	0.264
**Total QRS Power (%)**	31.857 (3.413)	29.489 (2.68)	0.607	30.104 (2.29)	31.876 (4.33)	0.728
**Total ST Power (Absolute)**	5.322 (2.92)	1.254 (1.092)	0.223	2.124 (1.149)	3.681 (2.593)	0.597
**Total ST Power (%)**	14.286 (11.318)	29.552 (11.546)	0.372	27.744 (10.331)	12.666 (13.757)	0.401
Attenuation observed in right precordial leads						
**Peak Power (Absolute)**	0.649 (0.286)	0.373 (0.081)	0.384	0.398 (0.105)	0.529 (0.188)	0.559
**Peak Power (%)**	23.7 (6.817)	16.195 (5.507)	0.419	22.376 (4.024)	3.07 (11.693)	0.136
**Total Power (Absolute)**	29.826 (13.237)	11.099 (3.466)	0.202	15.433 (4.897)	19.317 (8.213)	0.696
**Total Power (%)**	13.812 (5.69)	7.053 (6.675)	0.465	10.62 (5.634)	6.46 (7.582)	0.673
**Total QRS Power (Absolute)**	18.156 (9.234)	9.351 (1.875)	0.383	11.235 (3.308)	11.308 (4.145)	0.989
**Total QRS Power (%)**	25.677 (5.099)	17.772 (4.456)	0.271	22.454 (3.465)	11.235 (8.374)	0.235
**Total ST Power (Absolute)**	11.67 (6.401)	1.748 (2.53)	0.178	4.199 (2.738)	8.009 (5.317)	0.54
**Total ST Power (%)**	38.826 (20.559)	50.55 (16.648)	0.676	47.464 (15.303)	35.957 (21.692)	0.677

Table S4. Attenuation of the high frequency content after Ic drug infusion. Columns labeled as “Flecainide” and “Ajmaline” denote attenuation observed in the whole population (Induct-BrS plus NR). Units for Peak Power, Total Power, Total QRS Power and Total ST Power are expressed as $10^3 nV^2Hz^{-1}$. NR: Negative responders.

Table S5.

	BrS patients with Clinical Events (N=14)	Asymptomatic BrS patients (N=141)	p value
Clinical Features			
Age (years)	42.46 (34.36-50.56)	43.77 (41.46-46.08)	0.744
Male gender (%)	11 (78.57)	98 (69.5)	0.688
Family history of SCD at age <45 years (%)	6 (42.86)	80 (56.74)	0.475
Syncope (%)	9 (64.29)	30 (21.28)	**0.001**
Cardiac syncope (%)	8 (57.14)	11 (7.8)	**<0.001**
SCA (%)	14 (100)	0 (0)	**<0.001**
Smoker (%)	2 (14.29)	39 (27.66)	0.445
Hypertension (%)	3 (21.43)	22 (15.6)	0.854
Diabetes mellitus (%)	0 (0)	5 (3.55)	1
Dyslipidaemia (%)	1 (7.14)	29 (20.57)	0.391
Cardiomyopathy (%)†	0 (0)	6 (4.26)	1
Cardiovascular drugs (%)‡	3 (21.43)	26 (18.44)	0.863
PES Test performed	1 (7.14)	62 (43.97)	**0.017**
Positive PES	0 (0)	12 (8.51)	0.54
ICD implanted	13 (92.86)	32 (22.7)	**<0.001**
ECG pattern at the time of the digital record			
BrS type 1 (%)	5 (35.71)	33 (23.4)	0.487
BrS type 2 (%)	6 (42.86)	56 (39.72)	1
BrS type 3 (%)	1 (7.14)	21 (14.89)	0.696
BrS type 2-3 (%)	7 (50)	77 (54.61)	0.961
Normal (%)	2 (14.29)	23 (16.31)	1
High Frequency Content : All precordial leads			
Peak Power	1.1 (-0.16-2.37)	1.25 (0.44-2.07)	0.836
Total Power	79.53 (15.51-143.56)	55.28 (29.84-80.73)	0.465
Total QRS Power	28.35 (-4.22-60.93)	30.96 (10.7-51.22)	0.888
Total ST Power	51.18 (4.37-97.99)	24.32 (14.86-33.79)	0.248
QRS to ST Total Power	1.75 (0.85-2.65)	6.01 (4.51-7.51)	**<0.001**
High Frequency Content : Right precordial leads			
Peak Power	1.38 (-0.04-2.81)	1.48 (0.58-2.39)	0.903
Total Power	150.17 (2.58-297.77)	89.67 (51.24-128.09)	0.41
Total QRS Power	36.65 (-0.23-73.54)	40.56 (16.57-64.56)	0.854
Total ST Power	113.52 (-14.5-241.54)	49.1 (25.17-73.04)	0.307
QRS to ST Total Power	1.09 (0.58-1.59)	4.38 (3.24-5.53)	**<0.001**

Table S5. Comparative analysis of the clinical variables and the High Frequency Content between BrS patients with and without clinical events. Within brackets is denoting the

CI95%. Units for Peak Power, Total Power, Total QRS Power and Total ST Power are expressed as $10^3 nV^2 Hz^{-1}$. BrS: Brugada Syndrome.

Supplemental Figures

Figure S1

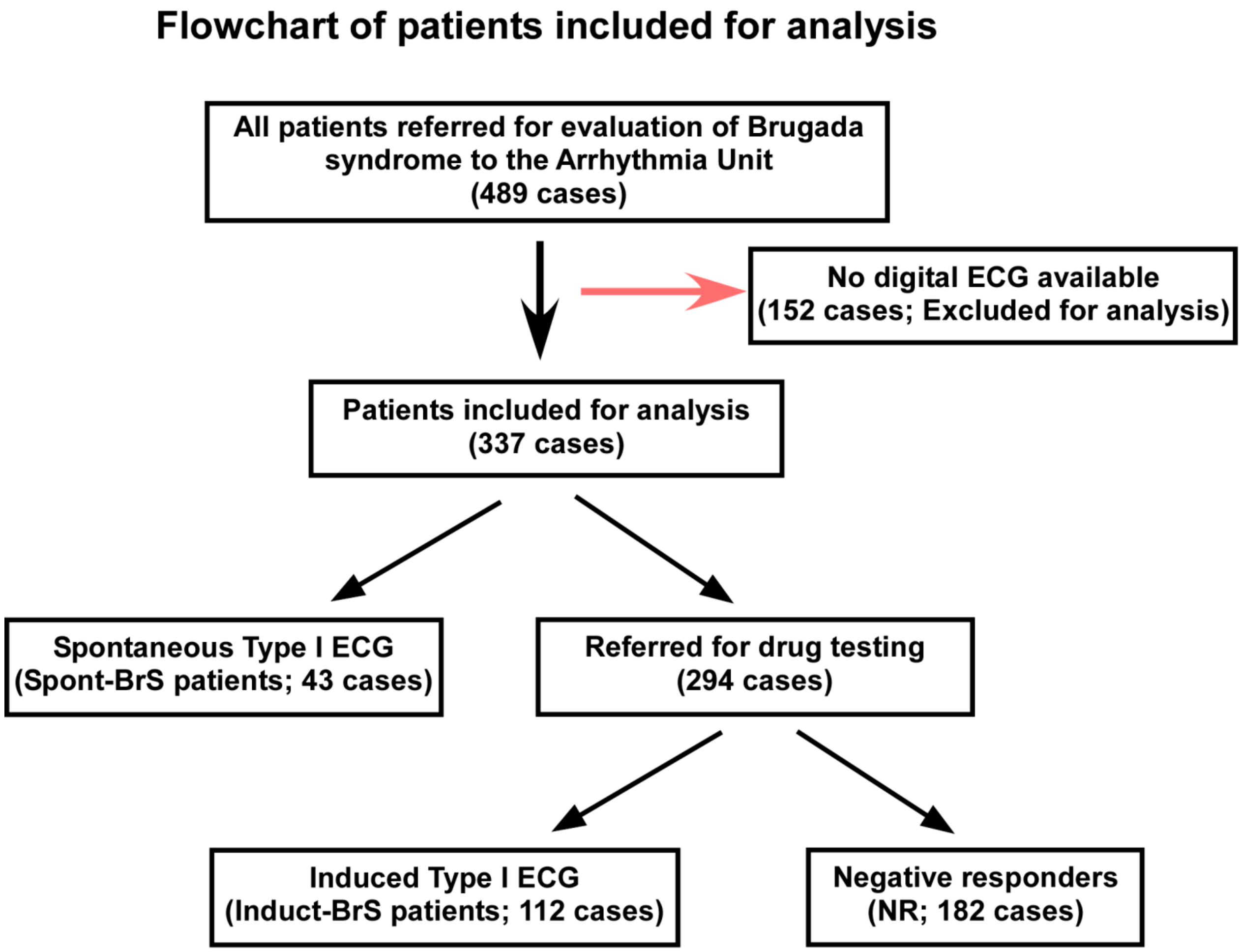

Figure S1. Flow-chart of patients included for analysis

Figure S2

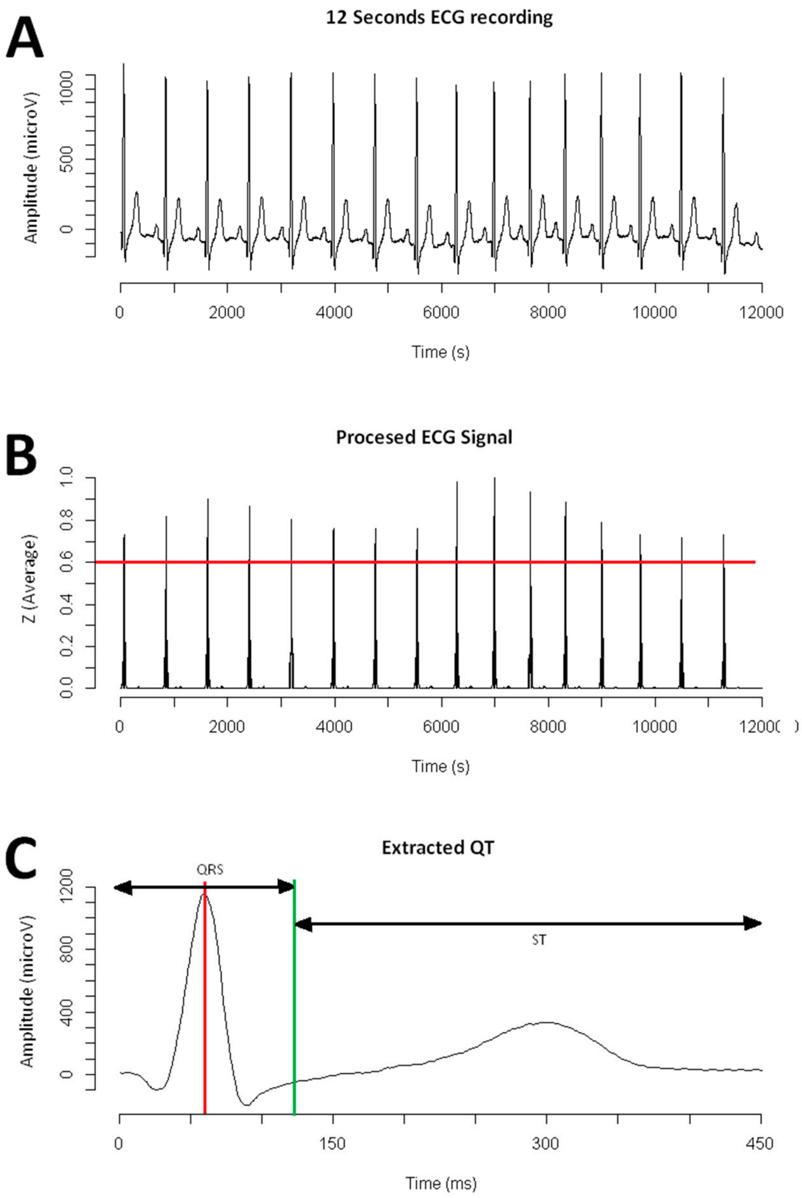

Figure S2. Example of the ECG processing and QT extraction. **Panel A:** Surface ECG (12 consecutive seconds). **Panel B:** Signal after derivation, square elevation and typification. The 0.6 threshold to detect the QRS complexes is marked (red line). **Panel C:** Example of an extracted QT complex. The *tMaxQRS* is indicated by the red line. Separation between QRS and ST is indicated by the green line. ECG: electrocardiogram.
